# Novel method for the identification of circulating SARS-CoV-2 variants and clinical characteristics of patient infection with SARS-CoV-2 variants in Central China

**DOI:** 10.3389/fcimb.2025.1605198

**Published:** 2025-11-04

**Authors:** Youhua Yuan, Yiman Geng, Tingjun Rong, Baoya Wang, Xiaohuan Mao, Xiaohuan Zhang, Xiulei Zhang, Yuan Zhang, Peiming Zheng, Jing Zhao, Fengxia Guo

**Affiliations:** ^1^ Department of Laboratory, Henan Provincial People’s Hospital, Zhengzhou, Henan, China; ^2^ Department of Laboratory, People’s Hospital of Zhengzhou University, Zhengzhou, Henan, China; ^3^ Department of Laboratory, People’s Hospital of Henan University, Zhengzhou, Henan, China; ^4^ Department of Laboratory, Xinyang Municipal Central Hospital, Xinyang, Henan, China

**Keywords:** acute coronavirus disease 2019, SARS-CoV-2 variant, nested PCR, next generation sequencing, clinical characteristic

## Abstract

**Introduction:**

We established a reliable and cost-effective method for identifying severe acute respiratory syndrome coronavirus 2 variants circulating in central China and analysed the clinical characteristics of patients with acute coronavirus disease 2019 who were infected with these variants.

**Methods:**

The RNA of centrifuged and enriched samples was extracted and reverse transcribed into cDNA. cDNA was then analysed using a nested polymerase chain reaction amplification and Sanger sequencing method targeting specific mutations in the spike, *ORF1a*, and *N* genes. This was validated against next-generation sequencing, achieving 100% concordance.

**Results:**

Among 172 isolates, JN.1.18.2 was the most prevalent (52.9%, 91/172), followed by XDV.1 (25.0%, 43/172), JN.1.16 (20.9%, 36/172), and KP.2 (1.2%, 2/172), which was found in central China for the first time. Fever with cough (52.6%, 80/152) was the most common symptom and 59.9% (91/152) of patients had underlying conditions. JN.1.18.2-infected patients more frequently presented with double-lung computed tomography changes. A strong positive correlation was observed between the duration from hospital admission to the detection of SARS-CoV-2 variants and total hospitalisation duration.

**Discussion:**

The new method provides a reliable tool for variant detection, highlighting milder clinical presentations in patients with active infections. Long-term monitoring of variants and patient characteristics is essential for effective prevention and treatment strategies.

## 1 Introduction

Coronavirus disease 2019 (COVID-19) rapidly spread across countries, causing a global pandemic (Ali et al., 2024). Severe acute respiratory syndrome coronavirus 2(SARS-CoV-2), a positive-stranded RNA virus that causes COVID-19, undergoes continuous evolution owing to the lack of proofreading by its RNA replicase, leading to mutations during transmission. Key mutations can increase viral infectivity, accelerate transmission, and drastically alter epidemic dynamics (Altare et al., 2024). The World Health Organization designated certain strains as “variants of concern” in November 2024 owing to their increased transmissibility, shorter incubation periods, and higher disease severity, contributing to epidemic rebounds globally (Hageman and Alcocer Alkureishi, 2022; Meganck et al., 2024; Wei et al., 2024).

The Omicron variant (B.1.1.529), first reported in South Africa on November 24, 2021, was detected in 57 countries by December 8, 2024 (Dai et al., 2024). Omicron replicates 70 times faster in human bronchial tissue than Delta (B.1.617.2), providing a significant transmission advantage (Ma et al., 2022; ML et al., 2024). Strengthening surveillance of viral mutations, assessing biological characteristics of variants, and developing vaccines are critical priorities for global epidemic control (Chen et al., 2024; Duan et al., 2024; Planas et al., 2024a). In May 2021, China issued the COVID-19 Prevention and Control Plan (version 8), emphasising SARS-CoV-2 variant surveillance and vaccine efficacy assessment (Chen et al., 2020; Han and Wu, 2024).

Currently, metagenomic next-generation sequencing (mNGS) is the gold standard for identifying viral variants by sequencing the full viral genome (Donzelli et al., 2022). Although advances in sequencing technology have simplified operations, mNGS remains expensive owing to high equipment, chip, and reagent costs and lacks the rapid throughput needed for immediate variant detection (Tartanian et al., 2023). Alternative methods for variant identification have been proposed but often lack reproducibility (Carattini et al., 2023; Nasereddin et al., 2022; Shen et al., 2024; Specchiarello et al., 2023; Wacharapluesadee et al., 2023).

Henan Province, located in central China with a population of 99 million (2024), remains underexplored in terms of clinical data on SARS-CoV-2 variants. Henan Provincial People’s Hospital, a tertiary care centre in Zhengzhou with 6,000 beds, serves critically ill patients across the 17 cities in this region. However, no studies have analysed the clinical characteristics of patients infected with SARS-CoV-2 variants in this region.

In this study, we established a novel nested polymerase chain reaction (PCR) and Sanger sequencing method to target partial gene sequences of the spike, N, and ORF1a regions, enabling the identification of circulating SARS-CoV-2 variants. Additionally, we examined the clinical characteristics of patients infected with different variants in central China, offering valuable insights for clinicians and public health policymakers to enhance control and treatment strategies.

## 2 Materials and methods

### 2.1 Sample collection

In total, 172 samples from 152 patients with confirmed COVID-19 and 5 negative SARS-CoV-2 throat swab isolates were collected between June 1, 2024 and January 31, 2025. These samples were obtained from the Department of PCR at Henan Provincial People’s Hospital. Additionally, to evaluate the specificity of SARS-CoV-2 variant identification, 45 positive isolates of other common respiratory pathogens were included. These comprised 10 influenza A (Flu A) throat swabs, 5 influenza B (Flu B) throat swabs, 5 respiratory syncytial virus (RSV) throat swabs, 5 *Mycoplasma* throat swabs, and 5 *Mycobacterium tuberculosis* sputum samples, which were sourced from the microbiology laboratory between June 1, 2024 and January 31, 2025.

### 2.2 RNA extraction and reverse transcription

Throat swab samples (2.6 mL of virus reserve liquid) or sputum samples (1 mL) were centrifuged at 15,000 × *g* for 1.5 h or not centrifuged, respectively. A 300-μL aliquot was subjected to RNA extraction using a nucleic acid extraction kit (Zhijiang Ltd., Shanghai, China) with an automatic extraction system (EX3600, Shanghai ZJ Bio-Tech Co., Ltd., Shanghai, China). The final RNA pellet was dissolved in 50 μL of RNase-free water and 10 μL of extracted RNA was reverse-transcribed into cDNA using a reverse transcriptase PCR kit (Mighty Script Plus Mix, Sangon Biotech Co., Ltd., Shanghai, China) and thermal cycler (T100, Bio-Rad, Hercules, CA, USA).

### 2.3 Primer design and validation

**Figure 1:**
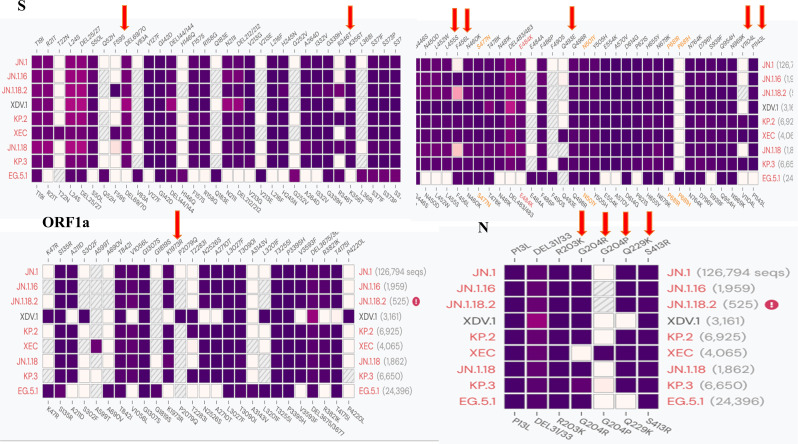
Comparison of different SARS-CoV-2 variants and specific mutation points in primer design.

**Figure 2:**
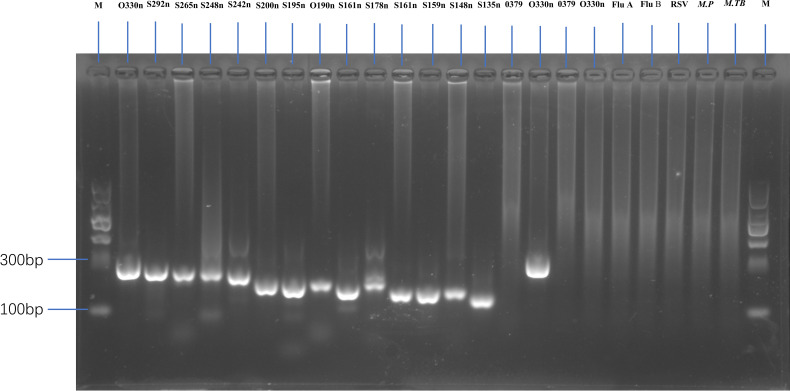
Gel electrophoresis showing amplification fragments of varying lengths by nested PCR. M, 1 to 24 panels represent DNA markers with fragment lengths of O330n, S292n, S265n, S248n, S242n, S200n, S195n, O190n, S161n, S178n, S161n, S159n, S148n, S135n, and O379 (first-round PCR product with centrifugation), O330 (second-round PCR product with centrifugation), O379 (first-round PCR product without centrifugation), and O330 (second-round PCR product without centrifugation) bp. Other lanes represent a negative SARS-CoV-2 sample, as well as Flu A, Flu B, RSV, *Mycoplasma pneumoniae (M.P)*, and *Mycobacterium tuberculosis (M. TB)* samples.

**Figure 3:**
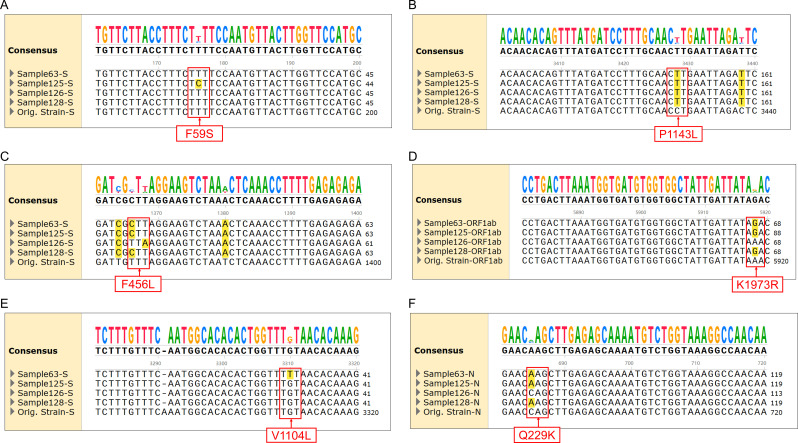
Alignment of sequencing results for specific mutations with different SARS-CoV-2 variants. **(A)** F59S mutation in the spike protein for JN.1.18.2 and XEC. **(B)** P1143L mutation in the spike protein for JN.1 series. **(C)** F456L mutations in the spike protein for JN.1 series and XDV.1. **(D)** K1973R mutation in ORF1a for JN.1 series. **(E)** V1104L mutation in the spike protein for KP.2, KP.3, and XEC. **(F)** Q229K mutation in the N protein for JN.1 series.

As most mutation points among SARS-CoV-2 variants are located in the spike, N, and ORF1a regions(https://gisaid.org/lineage-comparison/), in this study, genomic differences in the spike, N, and ORF1a regions among SARS-CoV-2 variants circulating in central China (June 2024 to January 2025) were analysed using online databases and https://ngdc.cncb.ac.cn/ncov/monitoring/country/China). Thirteen specific mutation points were selected (seven in the spike protein, four in the N protein, and one in the ORF1a protein) (**Figure 1**). Oligo 7 (version 7.60, Molecular Biology Insights, Toronto, Canada) was used to design seven nested PCR primer sets targeting these mutations based on the SARS-CoV-2 reference genome (MN908947, GenBank, Bethesda, MD, USA). Specificity was confirmed using NCBI Primer-BLAST (https://www.ncbi.nlm.nih.gov/tools/primer-blast/). Primer details are provided in **Table 1**. The specificity of primers was validated through nested PCR (**Figure 2**) and Sanger sequencing (**Figure 3**).

**Table 1:** Details of nested primers for identifying variants of SARS-CoV-2.

Primer name	Amplifying area and position	Direction	Sequence (5′-3′)	Annealing temperature	Product length	Specific point of variants
O379	ORF1a5768-6166	F	CAAGCTTCGATAATTTTAAGTTT	50	379	K1973R
		R	TAGGATTTTCCACTACTTCT			
O330n	ORF1a5793 -6142	F	ATGTGATAATATCAAATTTGCT	50	330	
		R	GACTGGTTTTAGATCTTCG			
O268	ORF1a5752-6019	F	CAACCATATCCAAACGCAAG	50	268	K1973R
		R	TATTTGGTTTATACGTGGCT			
O190n	ORF1a5794-5983	F	TGTGATAATATCAAATTTGCTG	50	190	
		R	TAACATGCCAAACAATAGGT			
O220	ORF1a5787-5807	F	GTTTGTATGTGATAATATCAAA	48	220	K1973R
		R	TGTTAACATGCCAAACAAT			
O156n	ORF1a5811-5967	F	TGCTGATGATTTAAACCAGT	50	156	
		R	GGTTTATGTAACAATTTAGCTC			
S334	S83-416	F	TACACTAATTCTTTCACAC	48	334	F59S
		R	AAAATGGATCATTACAAA			
S292n	S105-396	F	TGTTTATTACCCTGACAAA	50	292	
		R	TTGAAATTCACAGACTTT			
S229	S81-309	F	TACACTAATTCTTTCACACG	48	229	F59S
		R	TGTTAGACTTCTCAGTGGAA			
S161n	S103-263	F	GTGTTTATTACCCTGACAAA	50	161	
		R	CATCATTAAATGGTAGGACA			
S202	S81-283	F	ATACACTAATTCTTTCACACG	48	202	F59S
		R	GGAAGCAAAATAAACACCATC			
S135n	S103-238	F	GGTGTTTATTACCCTGACA	50	135	
		R	AAACCTCTTAGTACCATTGGTC			
S220	S1331-1551	F	GCATAGTGGTAATTATGAT	48	220	Q493E
		R	TAGAAGTTCAAAAGAAAGTAC			
S178n	S1352-1530	F	ACTGGTATAGATCGCTTAGG	50	178	
		R	ACTACTCTGTATGGTTGGT			
S300	S1262-1561	F	ATAATTATAAATTACCAGATGA	48	300	L455S F456L Q493E
		R	GTGCATGTAGAAGTTCAAA			
S248n	S1287-1534	F	TACAGGCTGCGTTATAGCTT	48	248	
		R	CTACTACTCTGTATGGTTGG			
S210	S1235-1444	F	CAGGGCAAACTGGAAA	50	210	L455S F456L
		R	CTTTACAAGGTTTGTTACCG			
S159n	S1265-1423	F	ATTATAAATTACCAGATGATT	50	159	
		R	CCTGATAGATTTCAGTTGA			
S280	S1276-1556	F	CCAGATGATTTTACAGG	48	280	L455S, F456L, Q493E
		R	GTAGAAGTTCAAAAGAAAG			
S242n	S1294-1536	F	TGCGTTATAGCTTGGAA	50	242	
		R	ACTACTACTCTGTATGGT			
S301	S3218-3517	F	AGAACTTCACAACTGC	48	301	P1143L, V1104L
		R	TAATGCCAGAGATGTCA			
S265-1n	S3235-3499	F	CCTGCCATTTGTCATG	48	265	
		R	CTAAATCAACATCTGGT			
S265	S3242-3506	F	TTTGTCATGATGGAAAAGCAC	48	265	P1143L
		R	ATGTCACCTAAATCAACATCTG			
S200n	S3264-3463	F	CTTTCCTCGTGAAGGTGTC	48	200	
		R	ATTTATCTAACTCCTCCTTGAA			
S260	S928-1188	F	AAGGAATCTATCAAACTTC	48	260	R346T
		R	ATAGACATTAGTAAAGCAGA			
S216n	S949-1164	F	AACTTTAGAGTCCAACCAA	48	216	
		R	ATTTAATTTAGTAGGAGACAC			
S250	S933-1182	F	AATCTATCAAACTTCTAACTT	50	250	R346T
		R	ATTAGTAAAGCAGAGATCATT			
S195n	S956-1150	F	GAGTCCAACCAACAGAATC	50	195	
		R	GAGACACTCCATAACACTT			
N343	N536-878	F	GCAGTCAAGCCTCTTCTCGTT	50	343	G204R G204P Q229K
		R	CTGATTAGTTCCTGGTCCCCAA			
N280n	N561-840	F	CTCATCACGTAGTCGCAACAG	50	280	
		R	TGGACCACGTCTGCCGAAA			

### 2.4 Nested PCR amplification of Spike, N, and ORF1a regions

A standard nested PCR protocol was developed to amplify the spike, N, and ORF1a regions of SARS-CoV-2. In the first round, 4 μL of template cDNA was amplified using 2× SanTaq PCR mixture (Sangon Biotech Co, Shanghai, China) in a thermal cycler (T100, Bio-Rad, Hercules, CA, USA). The thermal cycling parameters were as follows: pre-denaturation at 94°C for 5 min, 60 cycles of denaturation at 94°C for 30 s, annealing at 48°C for 30 s, and extension at 72°C for 18 s, followed by a final extension at 72°C for 10 min. For the second round, 10 μL of the first-round product was used as the template, with the same thermal cycling conditions except for a different denaturation temperature (50°C).

The nested PCR products were analysed via 1.5% agarose gel electrophoresis at 110 V for 35 min (**Figure 2**).

### 2.5 Sanger sequencing of SARS-CoV-2 Spike, N, and ORF1a protein partial genomes

Positive nested PCR products were subjected to Sanger sequencing at Shanghai Sangon Biotech (Shanghai, China) to analyse the partial genomes of the targeted spike, N, and ORF1a regions using the corresponding forward primer sets listed in **Table 1**.

### 2.6 Validation of the Sanger sequencing strategy for SARS-CoV-2 variant screening

To validate the accuracy of SARS-CoV-2 variant identification through nested amplification, 16 positive samples were analysed via NGS performed by Shanghai Sangon Biotech (Shanghai, China) and Zhengzhou Auto Diag Biotech (Zhengzhou, China) (**Table 2**). Whole-genome sequences were uploaded to the PANGOLIN platform (https://pangolin.cog-uk.io/) for variant identification.

**Table 2:** Identification criteria of SARS-CoV-2 variants by specific mutation points of the Spike protein, ORF1a, and N protein.

Variants	Spike								ORF1a	N			
	F59S	L455F	L455S	F456L	Q493E	R346T	V1104L	P1143L	K1973R	R203K	G204R	G204P	Q229K
JN.1.18.2	yes	no	yes	yes	no	yes	no	yes	yes	yes	yes	no	yes
JN.1.18	no	no	yes	yes	no	yes	no	yes	yes	yes	yes	no	yes
JN.1	no	no	yes	no	no	no	no	yes	yes	yes	yes	no	yes
JN.1.16	no	no	yes	yes	no	no	no	yes	yes	yes	yes	no	yes
KP.2	no	no	yes	yes	no	yes	yes	yes	yes	yes	yes	no	yes
KP.3	no	no	yes	yes	yes	no	yes	yes	yes	yes	yes	no	yes
XDV	no	no	yes	no	no	no	no	yes	no	yes	yes	no	no
XDV.1	no	no	yes	yes	no	no	no	yes	no	yes	yes	no	no
XEC	yes	no	yes	yes	yes	no	yes	yes	yes	yes	no	yes	yes
EG.5.1	no	yes	no	yes	no	no	no	no	no	yes	yes	no	no
HK.3	no	yes	yes	yes	no	no	no	no	no	yes	yes	no	no

### 2.7 Classification of notable SARS-CoV-2 variants

**Figure 4:**
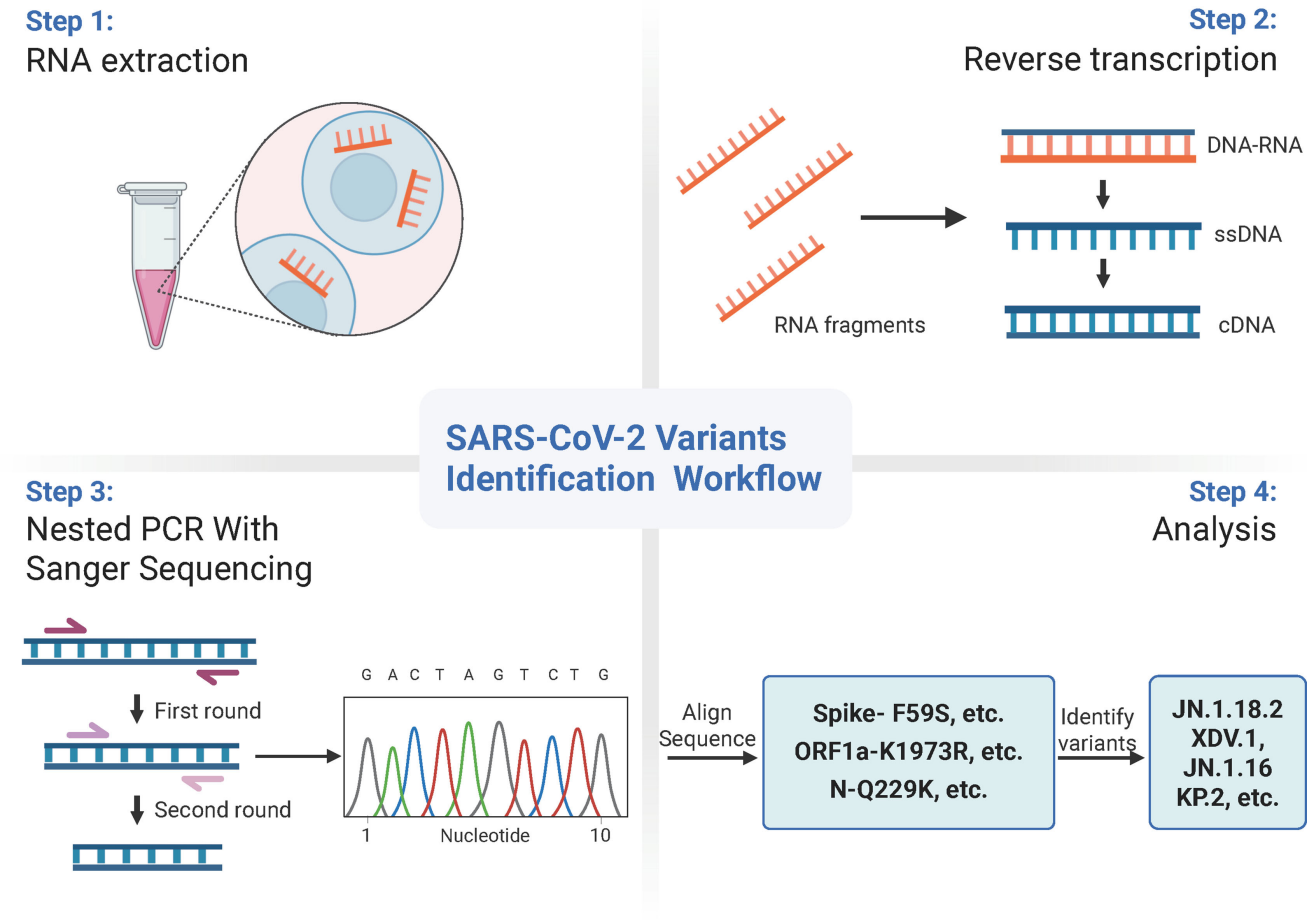
Schematic workflow for identification of SARS-CoV-2 variants.

Classification criteria for notable SARS-CoV-2 variants are outlined in **Table 3**. Samples were first amplified using seven nested primer sets to generate longer fragments. For samples that failed to produce the expected longer fragments, alternative nested primer sets were used to generate shorter fragments. Twelve specific mutation points within these fragments were sequenced via Sanger sequencing and sequence alignment was conducted using SnapGene (version 6.02, GSL Biotech, Chicago, IL, USA). Mutations were compared against the reference SARS-CoV-2 genome (MN908947) from GenBank (https://www.ncbi.nlm.nih.gov/nuccore/MN908947) (**Figure 3**). By analysing combinations of these 13 mutation points (**Table 4**), notable SARS-CoV-2 variants, including those spreading in China (e.g., XDV.1, JN.18.2, KP.2), were identified. The schematic workflow is shown in **Figure 4**.

**Table 3:** Clinical characteristics of patients infected with different SARS-CoV-2 variants (N = 152 patients).

Characteristics	JN.1.18.2 (n=76)	XDV.1 (n=39)	JN.1.16 (n=35)	KP.2 (n=2)	Total (N = 152)	P*-*value
Sex						
Men	45	20	18	0	83	0.383
Women	31	19	17	2	69	
Age	67 (57.3–77.5)	65 (51–74)	60 (41–73)	39 (20–39)	66.5 (51.5–75)	0.091
Family location in Henan Province						
East	12	4	8	0	24	0.130
West	3	3	3	1	10	
South	14	2	1	0	17	
North	7	2	3	0	12	
Centre	40	28	20	1	89	
Signs and symptoms						
Fever	16	14	14	0	44	0.144
Cough	1	0	0	0	1	
Fever and cough	48	17	14	1	80	
None	11	8	7	1	27	
Lung CT change						
Single	7	3	1	0	11	0.045
Double	49	20	15	0	84	
None	20	16	19	2	57	
Hospitalised days	12 (7–24)	11 (7–20)	9 (6–13)	16 (9–16)	10 (6–18)	0.279
Days from admission to positive SARS-CoV-2 sample	5 (1–10)	4 (1–8)	2 (1–7)	7.5 (3–7.5)	4 (1–9)	0.368
Underlying diseases						
Hypertension	36	15	10	1	62	0.252
Diabetes	25	6	6	0	37	0.110
Cerebral fraction	15	9	4	0	28	0.594
Coronary heart disease	17	6	10	0	28	0.516
Number of underlying diseases						
0	23	20	17	1	61	0.443
1	25	7	6	1	39	
2	11	6	5	0	22	
3	13	5	7	0	25	
4	4	1	0	0	5	
Hospitalised ward						
Internal	49	30	19	2	100	0.318
Surgery	2	1	3	0	6	
ICU	17	3	6	0	26	
Fever clinic	8	5	7	0	20	
Classification of severity						
Mild	63	31	30	2	126	0.850
Severe	13	8	5	0	26	
Outcomes						
Treatment success	65	34	32	2	133	0.821
Treatment failure	11	5	3	0	19	
Ct value						
ORF1a gene	19.7 (16.1–23.3)	20.5 (15.5–23.6)	15.4 (13.7–23.0)	15.2 (10.3–15.2)	18.1 (14.3–21.9)	0.295
N gene	19.1 (15.5–22.9)	20.5 (14.3–23.5)	15.7 (13.1–21.4)	14.3 (9.4–14.3)	19.9 (14.8–23.0)	0.308

**Table 4:** Clinical characteristics of patients infected with different SARS-CoV-2 variants (N = 152 patients) (continued).

Characteristics	JN.1.18.2 (n=76)	XDV.1 (n=39)	JN.1.16 (n=35)	KP.2 (n=2)	Total (N = 152)	P-value
Laboratory detection results						
WBC count (10^9^/L) Median (p25, p75)	8.8 (6.7, 8.9)	6.8 (4.8, 9.3)	6.4 (3.5, 7.7)	5.2 (4.3, 7.4)	6.7 (4.9, 8.9)	0.148
Lymphocyte count (10^9^/L) Median (p25, p75)	0.9 (0.8, 1.6)	0.8 (0.3, 1.1)	1.3 (0.4, 1.5)	1.2 (0.7, 2.2)	0.9 (0.6, 1.4)	0.426
Platelet count (10^9^/L) Median (p25, p75)	256 (187, 320)	95 (60.8, 162)	208 (144, 248)	221 (181, 251)	180 (124, 238)	0.060
CRP (ng/mL) Median (p25, p75)	18.4 (13.8, 176)	39.8 (13.8, 71.7)	12.2 (3.5, 25.3)	17.2 (3.4, 31.9)	15.0 (4.1, 43.9)	0.676
Procalcitonin (ng/mL) Median (p25, p75)	0.7 (0.1, 1.4)	0.5 (0.2, 1.5)	0.1 (0.05, 0.1)	0.05 (0.02, 0.1)	0.11 (0.05, 0.5)	0.127
D-dimer (ng/mL) Median (p25, p75)	2.2 (0.9, 6.9)	2.1 (0.8, 4.6)	2.8 (0.2, 8.5)	0.7 (0.2, 1.2)	1.6 (0.6, 3.96)	0.080
IL-6 (ng/mL) Median (p25, p75)	22.5 (13.18, 73.0)	60.2 (9.8, 280)	42.7 (1.4, 181)	_	22.5 (5.8, 182)	0.215
Alanine transaminase (IU/mL) Median (p25, p75)	17.0 (12.8, 27.6)	13.0 (10.6, 52.9)	20.6 (12.4, 31.0)	9.9 (8.8, 38.8)	21.7 (12.4, 50)	0.189
Lactate dehydrogenase (IU/mL) Median (p25, p75)	226 (210, 355)	244 (203, 319)	217 (160, 278)	166 (160, 237)	221 (182, 259)	0.376

### 2.8 Clinical characteristics of hospitalised patients with COVID-19 who were infected with SARS-CoV-2 variants

Clinical data for 132 hospitalised patients and 20 patients hospitalised in a fever clinic with confirmed SARS-CoV-2 infection were retrieved from the electronic medical record system of our hospital (Hospital Information System). Variables collected included age, sex, city of residence in Henan Province, duration from admission to positive SARS-CoV-2 sample, underlying comorbidities, symptoms, treatment outcomes, cycle threshold (Ct) values for the *ORF1a* and *N* genes, hospitalisation duration, and computed tomography (CT) features. Additionally, one set of laboratory data, including white blood cell (WBC), lymphocyte, and platelet counts and C-reactive protein (CRP), procalcitonin, alanine aminotransferase, D-dimer, IL-6, and lactate dehydrogenase concentrations, was collected on the test date with the shortest interval from the date of a SARS-CoV-2-positive test result. These data were analysed to characterise clinical differences among patients infected with distinct SARS-CoV-2 variants.

### 2.9 Definition of treatment outcomes and severity

Owing to traditional Chinese death culture, family members do not want the patient to die in the hospital and hope that the patient dies at home. Therefore, as the family members usually take the patient home prior to death, especially in rural areas, the cause of death is not clear in the medical records. Accordingly, we had to define treatment outcomes based on the medical records prior to death: voluntary discharge and discontinuation of treatment were defined as treatment failure, whereas treatment response leading to discharge was defined as treatment success. Additionally, we classified the patients into mild and severe cases according to the latest Chinese protocols for COVID-19 diagnosis and treatment (Released by National Health Commission of People’s Republic of China & National Administration of Traditional Chinese Medicine on January 5, 2023, 2023).

### 2.10 Statistical analysis

Summary statistics are presented as median with interquartile range or mean with standard error, depending on data distribution. Statistical significance among groups was assessed using the Kruskal–Wallis test with Bonferroni adjustments for continuous variables. Categorical variables were analysed using Pearson’s χ² test or Fisher’s exact test based on the feature of data. Correlations between continuous variables were evaluated using Spearman’s rank correlation, with significance set at p<0.05. All statistical analyses were conducted using SPSS (version 25.0; IBM Corp., Armonk, NY, USA) and GraphPad Prism (version 8.0; La Jolla, CA, USA).

### 2.11 Ethical considerations

The study was approved by the Ethics Committee of Henan Provincial People’s Hospital (Approval No. 241225). Written informed consent was obtained from all participants at the beginning of the study. All procedures adhered to the guidelines of the Declaration of Helsinki.

## 3 Results

### 3.1 Basic characteristics of patients infected with different SARS-CoV-2 variants

In total, 172 throat swab samples were collected from 152 patients with confirmed acute COVID-19 cases between June 1, 2024 and January 31, 2025. Among the 152 patients, 83 were men and 69 were women (median age, 67 years). Of these, 132 patients were hospitalised across 20 different inpatient wards, whereas 20 were treated in the fever clinic.

Twenty-six patients (17.1%, 26/152) had infections that were classified as severe cases requiring ICU admission and five patients died. In contrast, 82.9% (126/152) of the cases were classified as mild, based on the latest Chinese guidelines for the diagnosis and treatment of COVID-19 (Released by National Health Commission of People’s Republic of China & National Administration of Traditional Chinese Medicine on January 5, 2023, 2023; Wu et al., 2023). No significant difference was observed in the incidence of severe disease among patients infected with the JN.1.18.2, XDV.1 and JN.1.16 variants (*p* = 0.249, **Table 3**).

The 152 cases were distributed across 17 cities in Henan Province, spanning all directions. Most patients (58.6%, 89/152) were from Zhengzhou, the provincial capital located centrally, followed by 17 (11.2%, 17/152) from the southern region of Henan Province. No significant differences in the distribution of SARS-CoV-2 variants were observed across regions in central China.

The most common symptom was fever with cough (52.6%, 80/152). Additionally, 59.9% (91/152) of the patients had at least one underlying disease. Lung CT showed single or bilateral abnormalities in 62.5% (95/152) of the patients, whereas 37.5% (57/152) had no CT abnormalities. The median hospitalisation duration was 11 (interquartile range [IQR]: 7–20) days and the median interval from admission to a positive SARS-CoV-2 test was 4 (IQR: 1–9) days. Detailed clinical characteristics of patients infected with different SARS-CoV-2 variants are provided in **Table 3**.

### 3.2 Sensitivity and specificity of nested PCR for identifying SARS-CoV-2 variants

Nested PCR was performed for all 217 samples using the same primer set and reaction parameters. Among the 172 confirmed SARS-CoV-2-positive samples, all (100%, 172/172) were successfully amplified and sequenced using this method, indicating a sensitivity rate of 100%. Conversely, no amplification products were detected for the 45 pathogen-positive but SARS-CoV-2-negative samples, confirming a specificity rate of 100% (45/45). Additionally, we compared the PCR results of samples with and without centrifugation. The amplification of expected fragments in first- and second-round PCR was more successful with centrifuged samples than with non-centrifuged samples (**Figure 2**, lane 15-18).

### 3.3 Concordance rate of SARS-CoV-2 variants identified by nested reverse transcription (RT)-PCR and NGS

**Figure 5:**
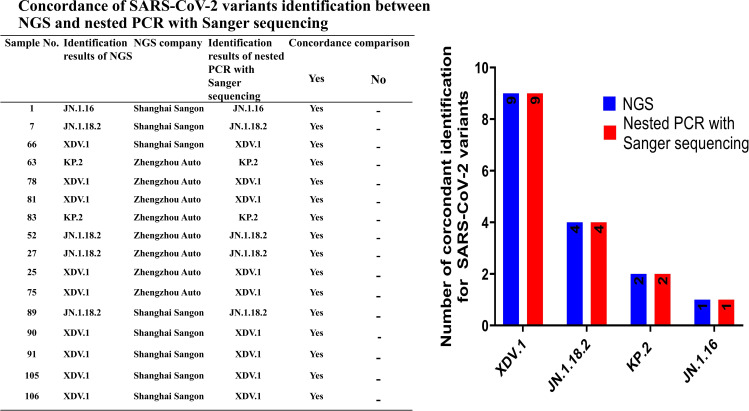
Concordance rate for identification of SARS-CoV-2 variants between NGS and nested PCR with Sanger sequencing.

We compared the results between NGS and nested PCR followed by Sanger sequencing, which showed 100% (16/16) concordance between the methods (**Figure 5**).

### 3.4 Distribution of notable SARS-CoV-2 variants

**Figure 6:**
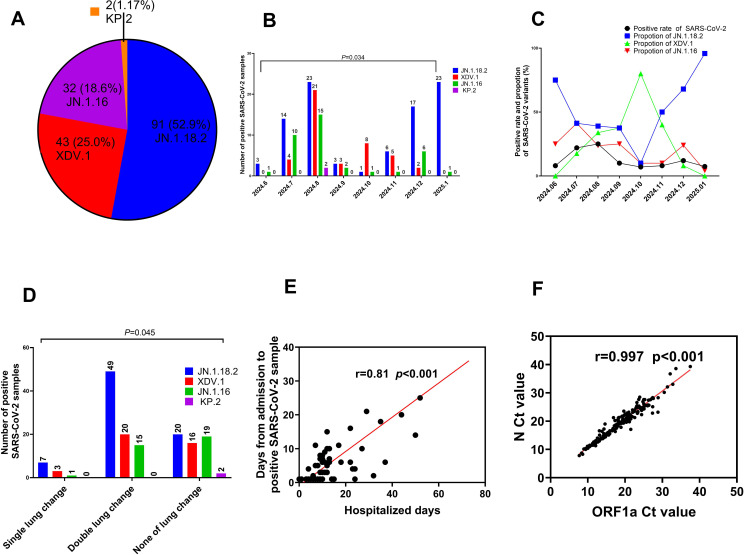
Comparison of key clinical characteristics among different SARS-CoV-2 variants. **(A)** Total proportion of different SARS-CoV-2 variants from June 2024 to January 2025. **(B)** Distribution of SARS-CoV-2 variants by month from June 2024 to January 2025. **(C)** Comparison of positive SARS-CoV-2 sample rates and constituent proportions of different variants from June 2024 to January 2025. **(D)** Comparison of age distribution among patients with COVID-19 who were infected with different SARS-CoV-2 variants. **(E)** Relationship between the Ct values of *ORF1a* and *N* gene. **(F)** Comparison of Ct values among patients with COVID-19 who were infected with different SARS-CoV-2 variants.

Among the 172 isolates screened, the most prevalent variant was JN.1.18.2, characterised by a specific F59S mutation in the spike protein (**Figure 3A**), accounting for 52.9% (91/172) of the isolates. This was followed by the XDV.1 variant (25.0%, 43/172) and JN.1.16 variant (20.9%, 36/172), which harboured specific L455S and F456L spike protein mutations. Additionally, a K1973R mutation in the ORF1a region was observed in the JN.1.16, JN.1.18.2, and KP.2 variants (**Figures 3B–D**). Two isolates were identified as variants of KP.2, which was found in Henan Province in central China for the first time, characterised by V1104L spike protein and Q229K N protein mutations (**Figures 3E, F**, **Figure 6A**).

The prevalence of SARS-CoV-2 and proportion of different variants varied significantly across months from June 2024 to January 2025. The highest positivity rate for SARS-CoV-2 infection occurred in August 2024, reaching 24.9% (59/237) (p=0.034, **Figure 6B**, **Supplementary Table S1**). In January 2025, the JN.1.18.2 variant accounted for 95.8% (23/24) of cases, representing its highest monthly proportion (**Figure 6C**).

### 3.5 Clinical characteristics of patients infected with SARS-CoV-2 variants

In total, 64.5% (49/76) of patients infected with the JN.1.18.2 variant exhibited bilateral lung abnormalities on CT, which was significantly higher than the 46.1% (35/76) observed in patients infected with other variants (*p* = 0.045, **Figure 6D**). Moreover, there was a strong positive correlation between the duration from hospital admission to the detection of SARS-CoV-2 variants and total hospitalisation duration (*r* = 0.81, *p* < 0.001, **Figure 6E**). Similarly, a strong positive linear correlation was observed between the Ct values of the *ORF1a* and *N* genes in patients infected with different SARS-CoV-2 variants (**Figure 6F**, *r* = 0.997, *p* < 0.001). Additionally, a significant positive linear relationship was found for the Ct values of ORF1a between patients infected with the XDV.1 and JN.1.16 variants (**Supplementary Figure S1A**, *r* = 0.64, *p* < 0.001).

**Figure 7:**
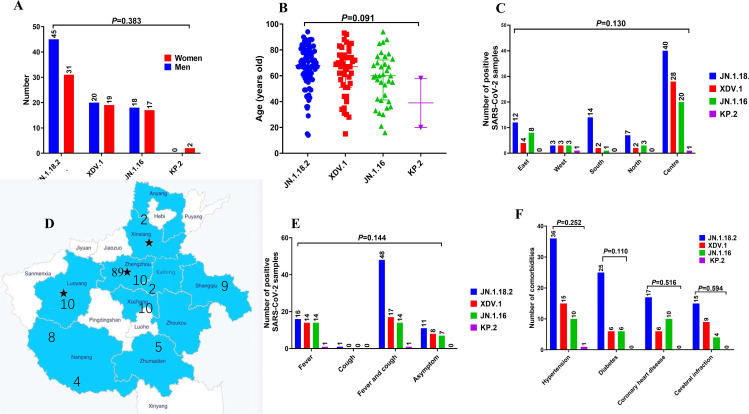
Comparison of primary clinical characteristics among patients infected with different SARS-CoV-2 variants. Comparison of **(A)** sex distribution, **(B)** age features, **(C, D)** different locations in central China, **(E)** signs and symptoms, and **(F)** comorbidities among patients with COVID-19 who were infected with different SARS-CoV-2 variants.

No significant differences were observed in the age or geographic location distribution among patients infected with different SARS-CoV-2 variants (p=0.091 and p=0.383, respectively, **Figures 7A–D**). Additionally, no significant differences were found in underlying comorbidities (hypertension, diabetes, coronary heart disease, or cerebral infarction) and clinical symptoms (fever or cough) among patients infected with different variants (**Figures 7E, F**). Similarly, no significant differences were observed in Ct values of the *ORF1a* and *N* genes (**Supplementary Figures S1B–F**, *p* = 0.110 and *p* = 0.594, respectively), disease severity classification, treatment outcome, ward distribution, number of comorbidities, hospitalisation duration, or time from admission to the detection of positive samples among patients infected with different SARS-CoV-2 variants (*p* = 0.279 to *p* = 0.850, respectively, **Supplementary Figures S2A–F**).

Laboratory detection results, including alanine aminotransferase concentration; WBC, lymphocyte, and platelet counts; and CRP, procalcitonin, D-dimer, IL-6, and lactate dehydrogenase concentrations, did not significantly differ among patients infected with different SARS-CoV-2 variants (*p* = 0.418 to *p* = 0.410, **Table 4**, **Supplementary Figures S3A–I**).

## 4 Discussion

In this study, we reported a novel nested PCR method for identifying SARS-CoV-2 variants circulating in central China and described the primary clinical characteristics of patients with acute COVID-19 who were infected with different variants. Our established method provides a reliable tool to monitor the evolutionary trends of SARS-CoV-2 within the population and serves as a reference for the development of vaccines targeting specific variants. Additionally, our findings on the clinical characteristics of different variants contribute valuable data for epidemic control measures and optimising the allocation of medical resources for patients with COVID-19. To the best of our knowledge, this is the first report to establish a nested PCR method for identifying circulating SARS-CoV-2 variants and describe the clinical characteristics of patients with acute COVID-19 who were infected with these variants in China.

Although several methods, including ordinary PCR sequencing, real-time quantitative PCR, and digital PCR, have been reported for identifying SARS-CoV-2 variants (Berno et al., 2022; Chhoung et al., 2024; Martínez-Murcia et al., 2023; Wu et al., 2023), our preliminary experiments revealed that many of the primers and protocols described in the literature were unable to amplify the expected PCR fragments. The primary limitation of previously reported methods was that their amplified fragments were often too long, reaching lengths of up to 600 base pairs (bp) (Martínez-Murcia et al., 2022). Another issue was that the primers used in these methods were often designed to amplify the variants prevalent in specific regions at the time of the studies. Over time, the composition of circulating variants has evolved and many primers now overlap with mutation points, rendering them ineffective for identifying current variants (Chhoung et al., 2024; Martínez-Murcia et al., 2022, 2023). To address these limitations, we self-designed our primers and established a robust nested PCR method capable of reliably identifying circulating SARS-CoV-2 variants in central China.

From our practical experience, the main challenges in clinical SARS-CoV-2-positive specimens include low viral loads and the poor integrity of extracted RNA templates. To overcome these issues, we enriched the virus content in the specimen by centrifugation at 15,000 × *g* for 1.5 h, increased the sensitivity of amplification, and developed a two-round nested PCR method with validated superiority in sensitivity and specificity. The key advantage of our method lies in its shorter amplification fragments: the maximum length was 330 bp (targeting the ORF1a site) and minimum length was 135 bp (targeting the spike protein site). We observed that shorter amplified fragments significantly increased the probability of successful amplification. To improve success rates, we designed alternative primer sets for each specific site, achieving a 100% identification success rate. This is also our first attempt to create a method of increasing virus concentration by centrifugation (Martínez-Murcia et al., 2022, 2023).

Unlike a previous study that focused solely on amplifying the spike protein to identify SARS-CoV-2 variants (Martínez-Murcia et al., 2022), our method incorporates additional mutation points in the ORF1a and N domains alongside those in the spike protein. This addition was crucial because we found that some variants, such as JN.1.16 and XDV.1, share identical spike protein mutations. Amplifying only the spike protein would have led to a failure in differentiating these two variants. This significant observation, which has not been reported in previous studies, underscores the importance of including multiple target regions for accurate variant identification.

Our results demonstrated that the proportion of the JN.1.18.2 variant in central China was higher than that of XDV.1, which contrasts with the most recent surveillance data released by the Chinese Center for Disease Control and Prevention (2025). According to the SARS-CoV-2 variant monitoring data from December 2024 by the Chinese CDC, the XDV.1 series accounted for a higher proportion (59.1%) across the entire country than the JN.1 series (37.6%), which includes the JN.1.18.2, JN.1.16, and KP.2 variants. This regional difference may be attributed to the specific geographic characteristics of Henan Province, which is centrally located in China. We identified two isolates of the KP.2 variant, which was previously prevalent in India and the United States (Kaku et al., 2024; Karyakarte et al., 2024; Kumar et al., 2024) but had only been found in limited quantities in coastal areas such as Guangdong, China. To the best of our knowledge, this is the first report of the KP.2 variant in Henan Province.

Regarding the clinical characteristics of 152 patients with COVID-19, our data showed that fever with cough was the most common symptom across infections with various SARS-CoV-2 variants. No significant difference was observed in signs and symptoms, mortality among severe cases, underlying conditions, treatment outcomes, nine laboratory detection results, or length of hospitalisation among patients infected with different variants. However, for older individuals with multiple underlying conditions, COVID-19 caused by various SARS-CoV-2 variants can still result in severe disease or even death (Planas et al., 2024b). Among the 26 patients with severe disease in our cohort, four died after infection with the JN.1.18.2 variant and one with the XDV.1 variant. Overall, as SARS-CoV-2 has evolved, the severity of COVID-19 has generally decreased, whereas the transmission potential of variants appears to be increasing. This trend is consistent with findings from other regions worldwide (Hughes et al., 2023; Kuthning et al., 2024).

Our findings revealed that patients infected with the JN.1.18.2 variant exhibited more double-lung CT changes than those infected with the XDV.1 and JN.1.16 variants. This observation has not yet been reported. We hypothesise that the JN.1.18.2 variant, with mutations such as F59S and L455S in the spike protein, may have a stronger immune escape capacity than the XDV.1 and JN.1.16 variants. This could explain why JN.1.18.2 is more likely to infect individuals with compromised immunity, such as older people and those with diabetes (Carey et al., 2022; Hasanzad et al., 2022; Liu et al., 2024). Additionally, we observed a strong correlation between the duration from admission to the detection of positive specimens and length of hospitalisation, suggesting that some patients acquired their infections in hospital settings. Given the recent shift in COVID-19 control measures from dynamic zero-COVID to routine epidemic control, infection rates among patients have increased compared with those in 2022 (Xu et al., 2024). This poses particular risks for older patients with underlying conditions. Routine epidemic controls highlight the need for strengthened infection control measures, particularly for hospitalised older patients, and education on self-protection measures, such as mask-wearing and maintaining social distancing. Furthermore, the development of a broad-spectrum COVID-19 vaccine is essential to address the continuous emergence of new SARS-CoV-2 variants (Chang et al., 2024; Link-Gelles et al., 2024; Subissi et al., 2024; Wu et al., 2022).

There are some limitations to our study. First, our research primarily focused on the identification of notable SARS-CoV-2 variants in China, which may have led to the exclusion of rare mutations or variants found in other regions, such as Africa and America (Aggarwal et al., 2023; Mboowa et al., 2024; Santoni, 2024; Zhao et al., 2022). Future studies will explore additional primers and real-time probe PCR methods to identify emerging SARS-CoV-2 variants globally. Second, our sample size and collection duration were relatively small. We plan to continue sample collection over a longer period to validate the reliability of our established method. Lastly, we will monitor genomic sequence trends, mutation patterns, and clinical manifestations of emerging variants to inform clinical strategies and policy decisions for COVID-19 control and treatment.

In conclusion, this study demonstrates the reliability and practicality of nested PCR with Sanger sequencing for screening samples for notable SARS-CoV-2 variants. Compared with NGS and other methods, our approach offers a cost-effective, universally applicable, and time-efficient tool for identifying emerging variants, particularly in regions where NGS may not be accessible. Through this method, we identified JN.1.18.2 and XDV.1 as the most prevalent SARS-CoV-2 variants in central China, with a smaller proportion of KP.2 variants. Moreover, patients infected with the JN.1.18.2 variant were more likely to have bilateral lung abnormalities on CT than those infected with the XDV.1 and JN.1.16 variants; therefore, early variant detection will enable clinicians to treat patients better, thereby avoiding severe COVID-19 and death. Although acute COVID-19 cases caused by these variants presented with milder symptoms than those caused by earlier strains, older patients with underlying conditions such as diabetes should be closely monitored for early diagnosis and treatment to prevent progression to severe disease.

## Data Availability

The original contributions presented in the study are included in the article/**Supplementary Material**. Further inquiries can be directed to the corresponding authors.
