# Supplementary material for: Novel method for the identification of circulating SARS-CoV-2 variants and clinical characteristics of patient infection with SARS-CoV-2 variants in Central China
Source: Front Cell Infect Microbiol. 2025 Nov 4;15:1605198. doi: 10.3389/fcimb.2025.1605198 (PMC12623389; doi:10.3389/fcimb.2025.1605198)
Supplement: Supplementary file 1 [file DataSheet1.docx]

Supplementary Material

# Supplementary Figures and Tables

## Supplementary Figures


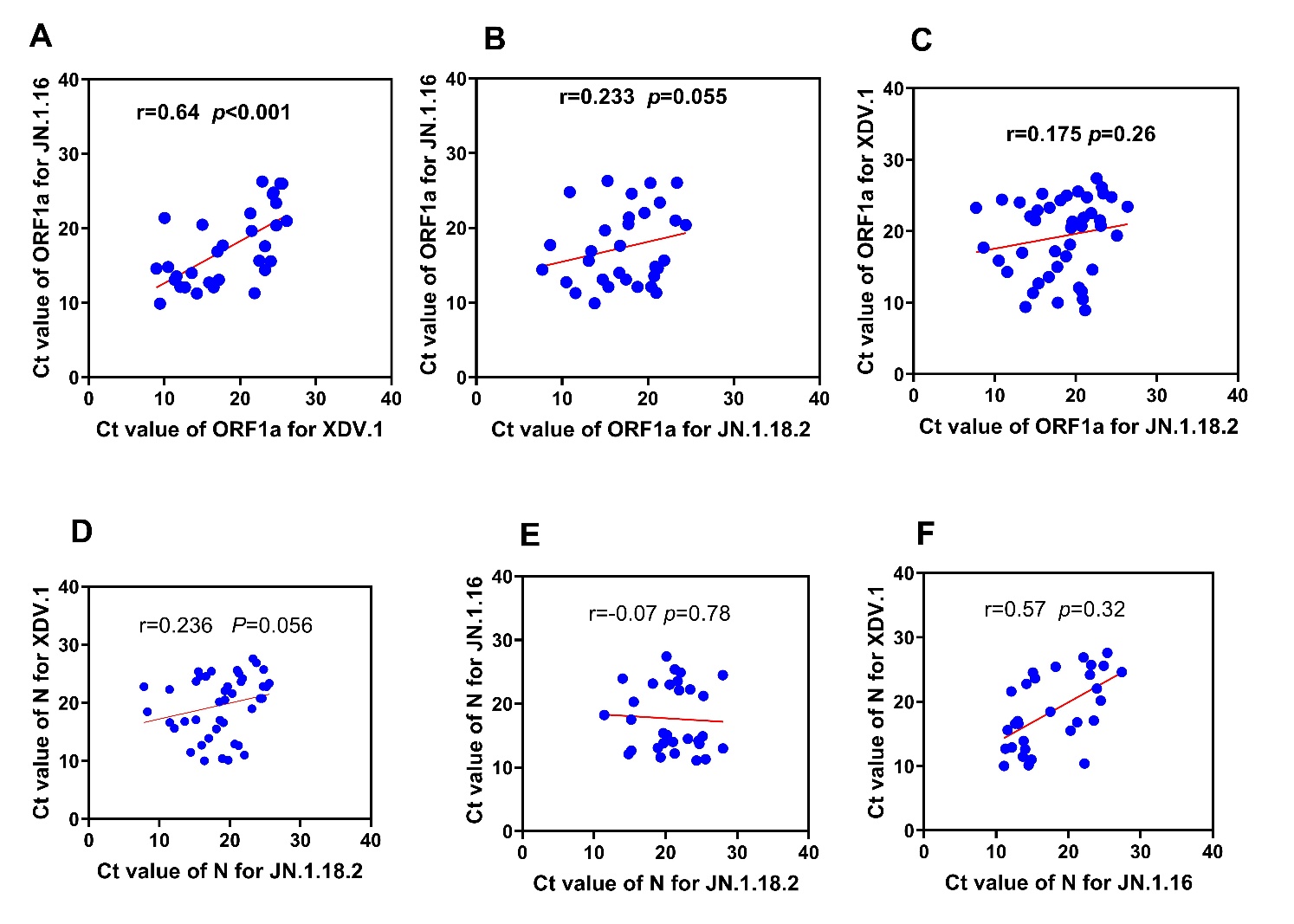


**Fig. S1**. Analysis of Ct values for the *ORF1a* and *N* genes among patients infected with different SARS-CoV-2 variants. Relationship between Ct values for the *ORF1a* gene in (**A**) XDV.1 and JN.1.16 variants, (**B**) JN.1.18.2 and JN.1.16 variants, and (**C**) JN.1.18.2 and XDV.1 variants and for the *N* gene in (**D**) JN.1.18.2 and XDV.1 variants, **(E**) JN.1.18.2 and JN.1.16 variants, and (**F**) XDV.1 and JN.1.16 variants among patients infected with different SARS-CoV-2 variants.


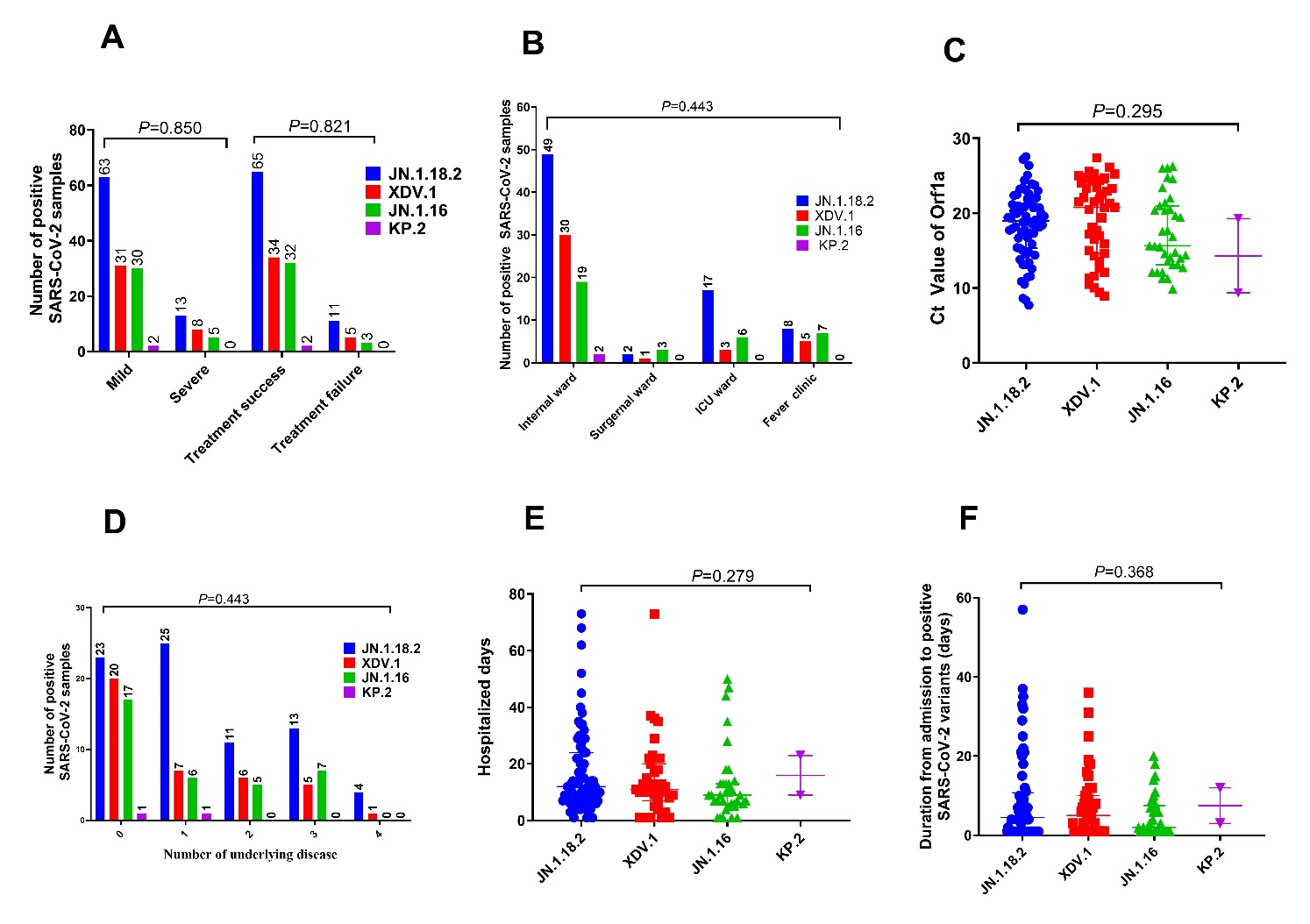


**Fig. S2**. Comparison of primary clinical characteristics among patients infected with different SARS-CoV-2 variants. Comparison of (**A)** case severity and treatment outcome, (**B**) ward assignment, (**C**) Ct value of ORF1a, (**D**) number of underlying diseases, (**E**) hospitalized days, and (**F**) duration from admission to positive SARS-CoV-2 detection among patients infected with different SARS-CoV-2 variants.


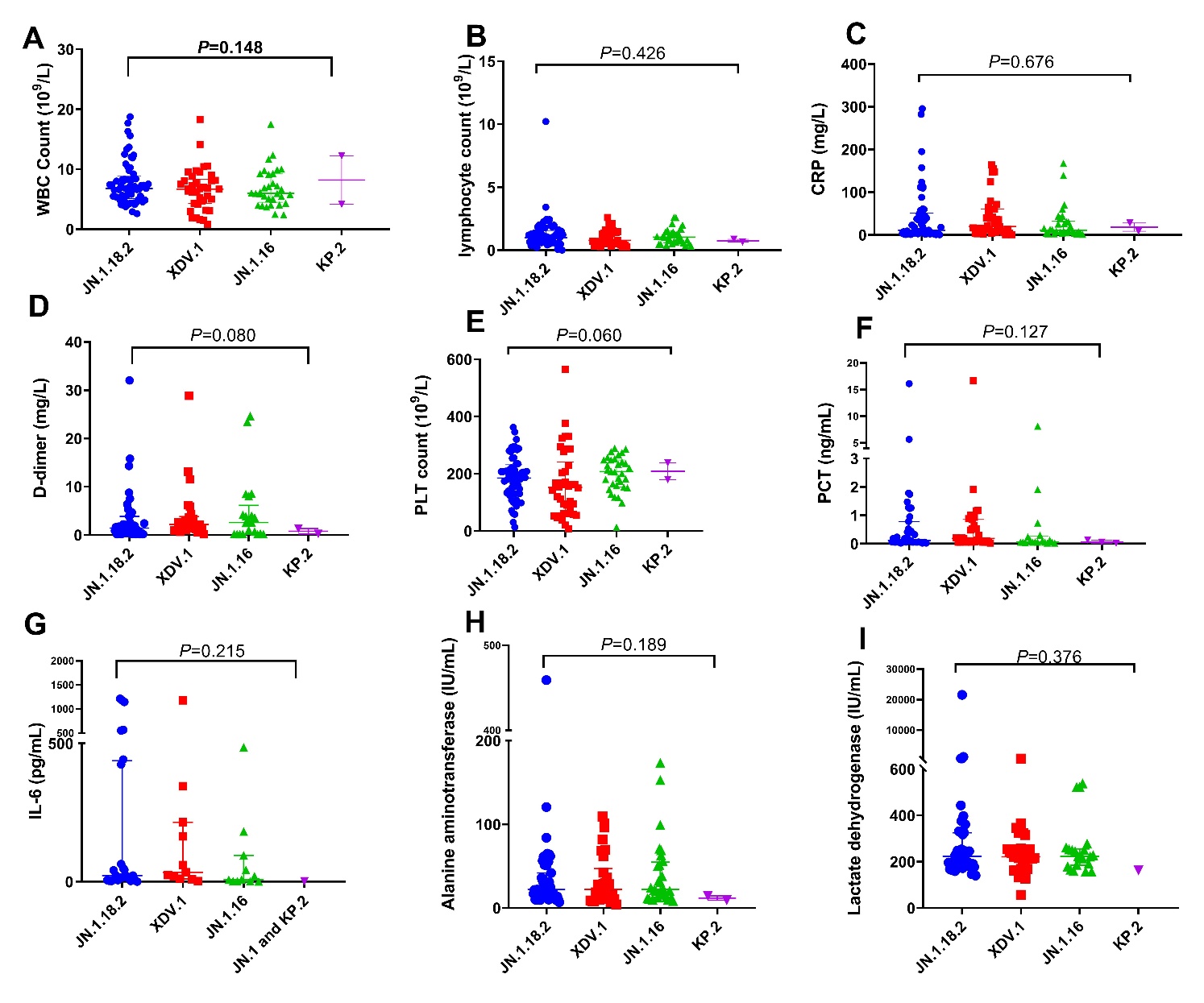


**Fig. S3**. Comparison of laboratory detection results among patients infected with different SARS-CoV-2 variants. Comparison of (**A**) WBC count, (**B**) lymphocyte count, (**C**) CRP concentration, (**D**) D-dimer concentration, (**E**) platelet count, (**F**) procalcitonin concentration, (**G**) IL-6 concentration, (**H**) alanine aminotransferase concentration, and (**I**) lactate dehydrogenase concentration among patients infected with different variants.

## Supplementary Tables

**Table S1.** Positivity rate of SARS-CoV-2 infection in inpatients and outpatients at Henan Provincial People’s Hospital

| Date | Jun 2024 | Jul 2024 | Aug 2024 | Sep 2024 | Oct 2024 | Nov 2024 | Dec 2024 | Jan  2025 | Total |
| --- | --- | --- | --- | --- | --- | --- | --- | --- | --- |
| Total number of people detected | 148 | 129 | 237 | 169 | 148 | 153 | 255 | 380 | 1619 |
| Number of SARS-CoV-2-positive individuals | 12 | 28 | 59 | 17 | 10 | 12 | 31 | 28 | 197 |
| Positivity rate of SARS-CoV-2 infection (%) | 8.1 | 21.7 | 24.9 | 10.1 | 6.8 | 7.8 | 12.2 | 7.4 | 12.2 |
